# CCL5 mediates breast cancer metastasis and prognosis through CCR5/Treg cells

**DOI:** 10.3389/fonc.2022.972383

**Published:** 2022-08-10

**Authors:** Juanjuan Qiu, Li Xu, Xiaohong Zeng, Hao Wu, Faqing Liang, Qing Lv, Zhenggui Du

**Affiliations:** ^1^ Department of Breast Surgery, West China Hospital, Sichuan University, Chengdu, China; ^2^ Key Laboratory of Transplant Engineering and Immunology NHC, West China Hospital, Sichuan University, Chengdu, China

**Keywords:** breast cancer, CCL5, CCR5, immune, Treg, metastasis

## Abstract

**Background and aims:**

CCL5 is considered to contribute to the biological function of a variety of cancer types, but its specific mechanism is still unclear. This study aimed to reveal the mechanism of CCL5 in the invasion, metastasis, and prognosis of breast cancer.

**Methods:**

The expression of CCL5 in tumor tissue and serum was measured with a Luminex protein detection kit, and the correlation between CCL5 and clinical parameters was evaluated. Kaplan–Meier analysis was used to analyze the effect of CCL5 on the prognosis of breast cancer patients. Protein interaction network analysis and gene coexpression were used to determine the receptor that has the strongest interaction with CCL5. Enrichment analysis was used to study the possible pathway by which CCL5 affects breast cancer progression. We used immunofluorescence staining and flow cytometry to estimate the fraction of immunity-related components in the tumor microenvironment.

**Results:**

The expression level of CCL5 in breast cancer patients was positively correlated with the degree of axillary lymph node metastasis; CCL5 in tumor tissue was correlated with estrogen receptor status (*P* = 0.034), progesterone receptor (*P* = 0.009), nuclear grade (*P* = 0.013), clinical stage (*P* < 0.001) and molecular subtype (*P* = 0.024) in breast cancer patients. Breast cancer patients with high CCL5 expression had worse disease-free survival (*P* = 0.031) and breast cancer-specific survival (*P* = 0.043); however, CCL5 had no effect on overall survival (*P* = 0.077). CCL5 affected tumor progression through CCR5, and the T-cell-related immune pathway may be the main pathway; the CD4+/CD8+, CCR5+/CD4+ and Treg/CCR5+ cell ratios were significantly increased in the lymph node metastasis group.

**Conclusion:**

CCL5 affects the Treg/CD4+CCR5+ cell ratio in breast cancer patients through CCR5, thus affecting breast cancer metastasis and prognosis.

## Introduction

According to data released by the World Health Organization Cancer Research Institute at the beginning of 2021, it is estimated that there are approximately 2.3 million new cases of breast cancer. For the first time, breast cancer surpassed lung cancer to become the most common cancer in the world, accounting for approximately 11.7% of new cancer cases (1). Even for patients diagnosed with early breast cancer at the time of initial diagnosis, there is still a 20%-30% chance of distant metastasis after treatment, and approximately 4%-6% of patients have distant metastasis at the time of diagnosis (2). Metastasis is the main cause of death of breast cancer patients, and it is very difficult to cure the disease once metastasis occurs.

The interaction between tumor cells and stromal cells in the tumor microenvironment is one of the key factors in tumor progression. Among them, immune cells (including tumor-associated macrophages, tumor-associated monocytes and lymphocytes) are important components of the stroma. Many studies have confirmed that tumor infiltrating lymphocytes (TILs) can significantly enhance the killing effect of immune cells on tumor cells, thus affecting the progression of tumors and the prognosis of cancer patients (3–5). However, another type of lymphocyte, regulatory T cells (Tregs), can differentiate into cells beneficial to the tumor itself during the “education” of cancer and then promote the growth and metastasis of tumor cells (6–9). Generally, these two heterogeneous lymphocyte types coexist in tumors and restrict each other. When the number of the former lymphocyte type, with killing effects on the tumor, increases and their function is enhanced, the tumor cells are killed, inhibited or grew slowly; in contrast, when the number and function of the other lymphocyte type are dominant, tumor cells easily escape the monitoring of immune cells and are more prone to invasion and metastasis.

Chemokine C-C motif ligand-5 (CCL5), also known as regulated upon activation and normal T-cell expressed and secreted, is expressed in T lymphocytes, macrophages, platelets, synovial fibroblasts, epithelial cells and specific types of cancer cells, of which T lymphocytes are the most important (10–12). CCL5 can mediate the migration and chemotaxis of inflammatory cells, including memory T lymphocytes (13), monocytes, and eosinophils (14). In addition, CCL5 is associated with the metastasis and prognosis of ovarian cancer, prostate cancer, pancreatic cancer and melanoma (15, 16). As a chemokine, the role of CCL5 depends on its binding with corresponding receptors (CCRs). Several studies have reported that CCL5 can combine with a variety of receptors, such as CCR1, CCR3, CCR4, CCR5 (17, 18), CD44 (17, 19) and GPR75 (20), to exert its biological functions.

Our previous study found that CCL5 in serum may be positively correlated with lymph node metastasis in breast cancer (21), but the source of CCL5 *in vivo* and the reasons for its correlation with lymph node metastasis are still unclear. Therefore, this study focused on the receptors and pathways related to the CCL5 protein to study the role and possible mechanism of CCL5 in the metastasis of breast cancer.

## Methods and patients

### Tissue specimen collection and follow-up of patients

**Figure 1:**
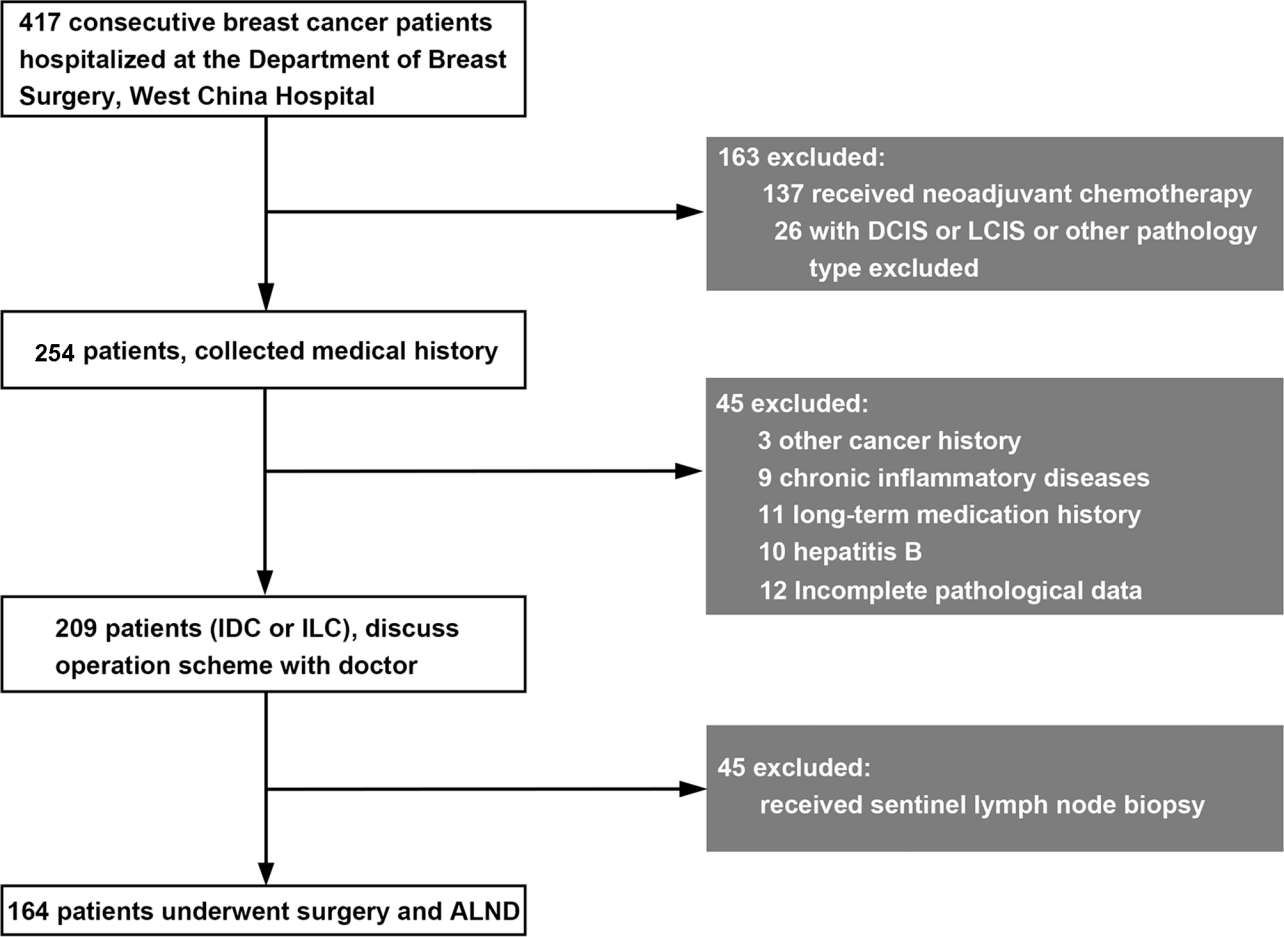
Inclusion and exclusion criteria for breast cancer patients in this study. DCIS, ductal carcinoma in situ; LCIS, lobular carcinoma in situ; IDC, invasive ductal carcinoma; ILC, invasive lobular carcinoma.

This study included 417 patients who underwent breast surgery at West China Hospital of Sichuan University from November 2017 to July 2018. The inclusion and exclusion criteria are shown in **Figure 1**. According to the inclusion and exclusion criteria, 164 patients were finally included. After written informed consent was obtained, tumor tissue and blood samples were collected. Each patient was followed up every year to ensure reliable prognostic information. The study was conducted in accordance with the Declaration of Helsinki, and the protocol was approved by the medical ethics committee of Sichuan University (approval No. [2018] 209).

### Extraction and detection of tissue and blood proteins

Tumor tissues and blood proteins were extracted, and 100 µl of frozen serum and lysate were transferred to 1.5 ml EP tubes. The final concentration (pg/ml) was obtained by following the operating instructions of the LumineX protein assay kit.

### Breast cancer patient microarray gene expression data from the Gene Expression Omnibus (GEO) database and The Cancer Genome Atlas (TCGA) database

The expression profile (GSE20194, GSE20271, GSE22093, GSE23988, GSE25066) was obtained from GEO (http://www.ncbi.nlm.nih.gov/geo/), a free and publicly available database based on the GPL570 and GPL96 platforms. The protein–protein interaction network analysis and enrichment analysis were based on the GEO database, and the coexpression analysis was based on TCGA database.

### Immunofluorescence staining and flow cytometry assay

Immunofluorescence was conducted as previously described (22). CD4, CD8, CCL5, and CCR5 were observed in breast cancer tissues. The slides were stained with 4’,6-diamidino-2-phenylindole for 5 min and viewed *via* fluorescence microscopy.

After isolation, antibody incubations were performed on ice for 30 min, and samples were washed twice with 1 mL phosphate-buffered saline. For membrane and intracellular staining, cells were incubated with anti-human CCR5, anti-human CCL5, and anti-human CD4 and CD8 antibodies. Flow cytometry data were analyzed with FlowJo software version 10 (Becton, Dickinson and Company, Franklin Lake, NJ, USA).

### Statistical analysis

Statistical analyses were performed using R statistical software version 3.5 with related packages or our customized functions and SPSS 25.0 software (IBM Corporation, Armonk, NY, USA). Data are presented as the mean ± standard deviation and were compared using Student’s test or analysis of variance. Survival curves were estimated using the Kaplan–Meier method, and the logarithmic rank test was used to assess the difference in survival curves. *P <*0.05 (two-sided) was considered statistically significant.

## Results

### Clinical characteristics of the included patients

According to the lymph node metastasis status of the patients, the included patients were divided into a nonmetastatic group, a 1-3 lymph node metastases group and a ≥ 4 lymph node metastases group. The clinical characteristics of the patients are shown in **Table 1**. Among 164 patients, approximately 51% had negative lymph nodes, and 19% had more than 3 metastatic lymph nodes. The positive rates of ER and PR were 75.6% and 72%, respectively.

**Table 1:** Clinical characteristics of 164 patients with breast cancer included in this study.

Parameters	(n) (%)	LN (–) (*n = 84*) (51.2%)	LN (1-3) (*n* = 49) (29.9%)	LN ≥ 4 (*n *= 31) (18.9%)
Age (years)		49.42 ± 9.67	46.86 ± 8.81	49 ± 8.63
Tumor size (cm)				
≤2	104 (63.4)	64 (76.2)	23 (46.9)	17 (54.8)
>2 and ≤5	53 (32.3)	18 (21.4)	23 (46.9)	12 (38.7)
>5	7 (4.3)	2 (2.4)	3 (6.1)	2 (6.5)
ER				
negative	40 (24.4)	20 (23.8)	12 (24.5)	8 (25.8)
positive	124 (75.6)	64 (76.2)	37 (75.5)	23 (74.2)
PR				
negative	46 (28)	25 (29.8)	11 (22.4)	10 (32.3)
positive	118 (72)	59 (70.2)	38 (77.6)	21 (67.7)
Her2				
negative	117 (71.3)	56 (66.7)	38 (77.6)	23 (74.2)
positive	47 (28.7)	28 (33.3)	11 (22.4)	8 (25.8)
Ki-67				
<14%	73 (44.5)	39 (46.4)	18 (36.7)	16 (51.6)
≥14%	91 (55.5)	45 (53.6)	31 (63.3)	15 (48.4)
Molecular subtype				
Luminal A	34 (20.7)	18 (21.4)	8 (16.3)	8 (25.8)
Luminal B	95 (57.9)	48 (57.1)	31 (63.3)	16 (51.6)
HER2-overexpressed	15 (9.1)	11 (13.1)	3 (6.1)	1 (3.2)
Triple-negative	20 (12.2)	7 (8.3)	7 (14.3)	6 (19.4)
Histological classification				
Low grade	20 (12.2)	9 (10.7)	7 (14.3)	4 (12.8)
Moderate grade	86 (52.4)	45 (53.6)	25 (51.0)	16 (51.2)
High grade	58 (35.4)	30 (35.7)	17 (34.7)	11 (36.0)

ER, estrogen receptor; PR, progesterone receptor; Her2, human epidermal growth factor receptor 2.

### The level of CCL5 in breast cancer patients is related to the axillary lymph node metastasis status

**Figure 2:**
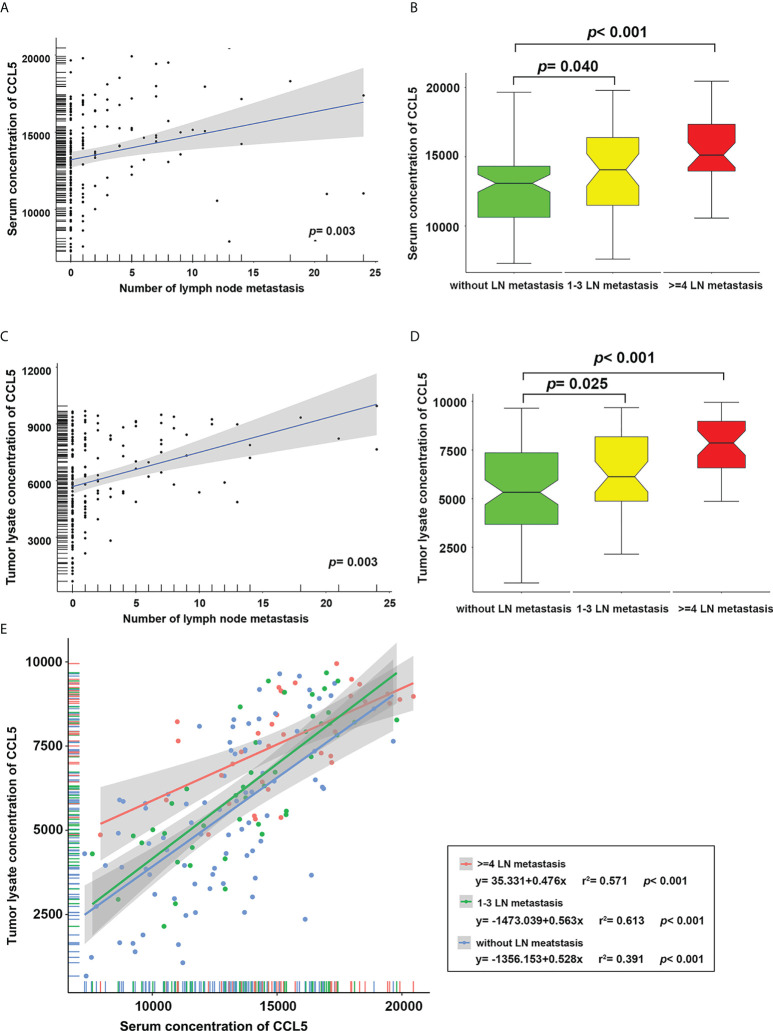
The level of CCL5 in serum **(A)** and tumor tissue lysate **(C)** was positively correlated with the number of lymph node metastases. The levels of CCL5 in serum **(B)** and tumor tissue lysate **(D)** in patients with 1-3 and ≥ 4 lymph node metastases were significantly higher than those in the lymph node-negative group. **(E)** Regardless of the number of axillary lymph node metastases, the level of CCL5 in serum was positively correlated with that in tumor tissue.

The expression levels of CCL5 in the serum and tumor tissue of breast cancer patients were detected with a Luminex protein detection kit, and it was found that the expression of CCL5 in serum was positively correlated with the number of axillary lymph node metastases in breast cancer patients (**Figure 2A**). The patients were further divided into three groups according to the number of lymph node metastases: a lymph node-negative group, a 1-3 lymph node metastases group and a ≥ 4 lymph node metastases group. The results showed that the serum CCL5 expression level in the lymph node 1-3 and ≥ 4 positive groups was higher than that in the lymph node-negative group, and the *P* values indicated significant differences (**Figure 2B**, *P* values were 0.04 and < 0.001, respectively). Similar results were found in the analysis of tumor tissue lysates (**Figures 2C, D**, *P* values were 0.025 and < 0.001, respectively).

In addition, linear regression analysis was carried out for CCL5 levels in serum and tumor tissue to explore the relationship between lymph node metastasis and CCL5. We found that regardless of the lymph node metastasis grouping, the level of CCL5 in serum was positively correlated with that in tumor tissue (**Figure 2E**, all *P* < 0.001); the regression equation is shown in **Figure 2E**.

### Analysis of correlations between CCL5 levels and clinicopathological features and survival in breast cancer patients

**Figure 3:**
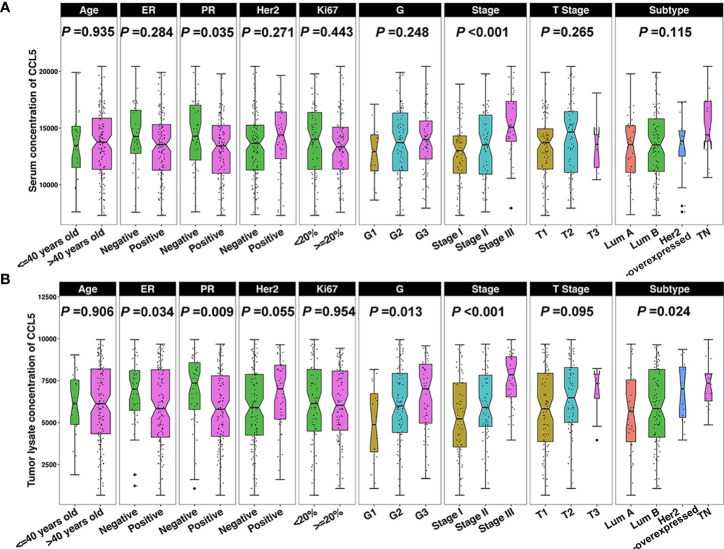
**(A)** The serum CCL5 concentration is correlated with PR and clinical stage, and **(B)** CCL5 levels in tumors are correlated with ER, PR, nuclear grade, clinical stage, and molecular subtype. ER, estrogen receptor; PR, progesterone receptor; Her2, human epidermal growth factor receptor; G, nuclear grade; Lum, luminal; TN, triple negative.

We conducted a correlation analysis in breast cancer patients to determine whether CCL5 levels are related to clinicopathological features. The results showed that CCL5 levels in serum were higher in PR-negative breast cancer (*P* = 0.035) and higher in breast cancer patients with late clinical stage disease (*P* < 0.001) (**Figure 3A**). However, CCL5 levels in tumor tissue were higher in breast cancer patients with ER-negative (*P* = 0.034) or PR-negative (*P* = 0.009) tumors, a higher nuclear grade (*P* = 0.013) and a later clinical stage (*P* < 0.001). In addition, CCL5 was highly expressed in HER2-overexpressing and triple-negative breast cancer subtypes (*P* = 0.024) (**Figure 3B**). In other words, CCL5 is positively correlated with clinicopathological features of breast cancer that predict poor prognosis and may also affect disease progression in breast cancer patients as a factor of poor prognosis in breast cancer.

**Figure 4:**
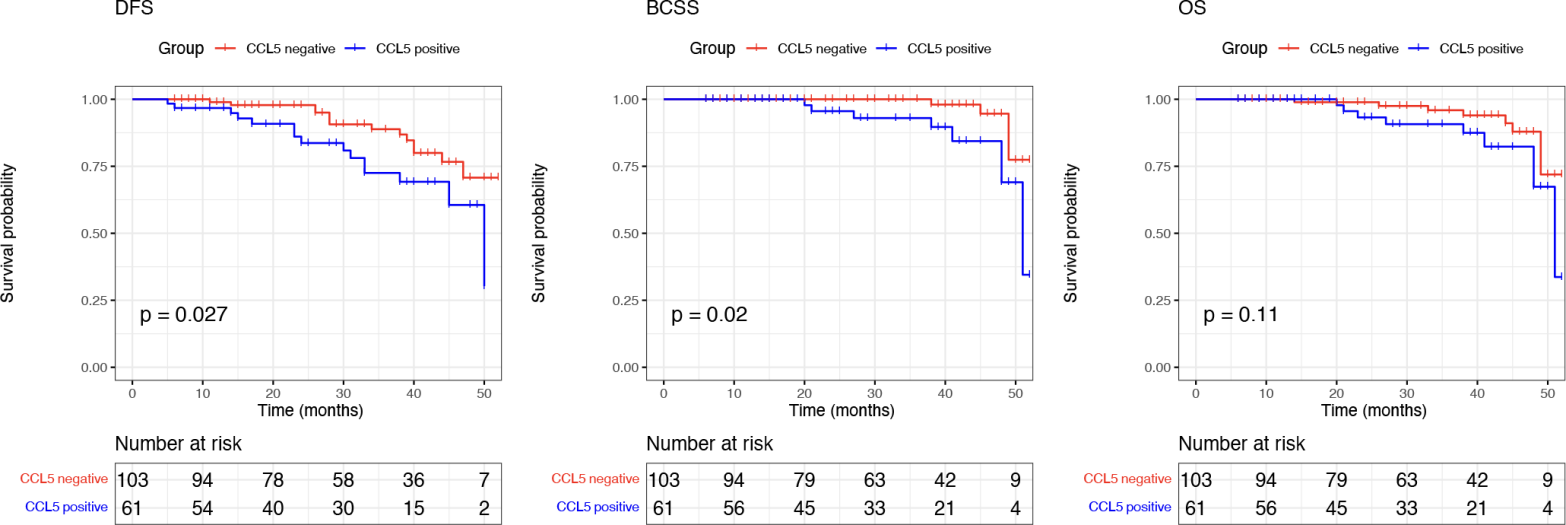
Breast cancer patients negative for CCL5 have better DFS and BCSS.

We performed Kaplan–Meier survival analysis based on the prognostic information of the included patients. The mean follow-up time was 30.07 (5-52 months), and the results showed that breast cancer patients negative for CCL5 had better DFS (*P* =0.027) and BCSS (*P* =0.013). There was no significant difference in OS between the two groups (*P* = 0.079) (**Figure 4**).

### CCL5 may promote breast cancer progression mainly through CCR5

**Figure 5:**
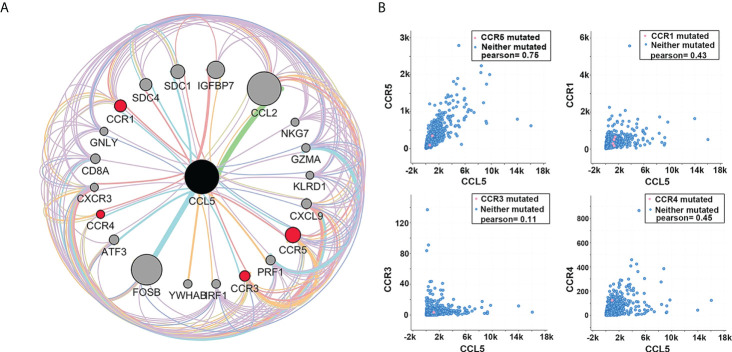
**(A)** Protein interaction network analysis shows that among the receptors of CCL5, the correlation between CCR5 and CCL5 is the strongest. The thicker the connecting line is, the larger the node, and the darker the color is, the greater the intensity of interaction. **(B)** Coexpression analysis showed that among several receptors with a strong interaction with CCL5, the coexpression of CCR5 and CCL5 was the strongest (Pearson = 0.75).

Based on the results of the previous analysis, we speculate that CCL5 may be an important factor affecting axillary lymph node metastasis and prognosis in patients with breast cancer. As a cytokine ligand, its role depends on the corresponding receptor. Through analysis of the GEO database (GSE20194, GSE20271, GSE22093, GSE23988, GSE25066), we found that CCR1, CCR3, CCR4 and CCR5 were highly correlated with CCL5, and among them, CCR5 showed the strongest correlation (**Figure 5A**).

We analyzed the coexpression of the above receptors with CCL5 in TCGA database and found that CCL5 and CCR5 had the strongest coexpression (Pearson =0.75), indicating that CCL5/CCR5 may act as a signaling pathway or complex that promotes breast cancer progression (**Figure 5B**).

### CCL5/CCR5 affects breast cancer metastasis through T-cell-related immune pathways

**Figure 6:**
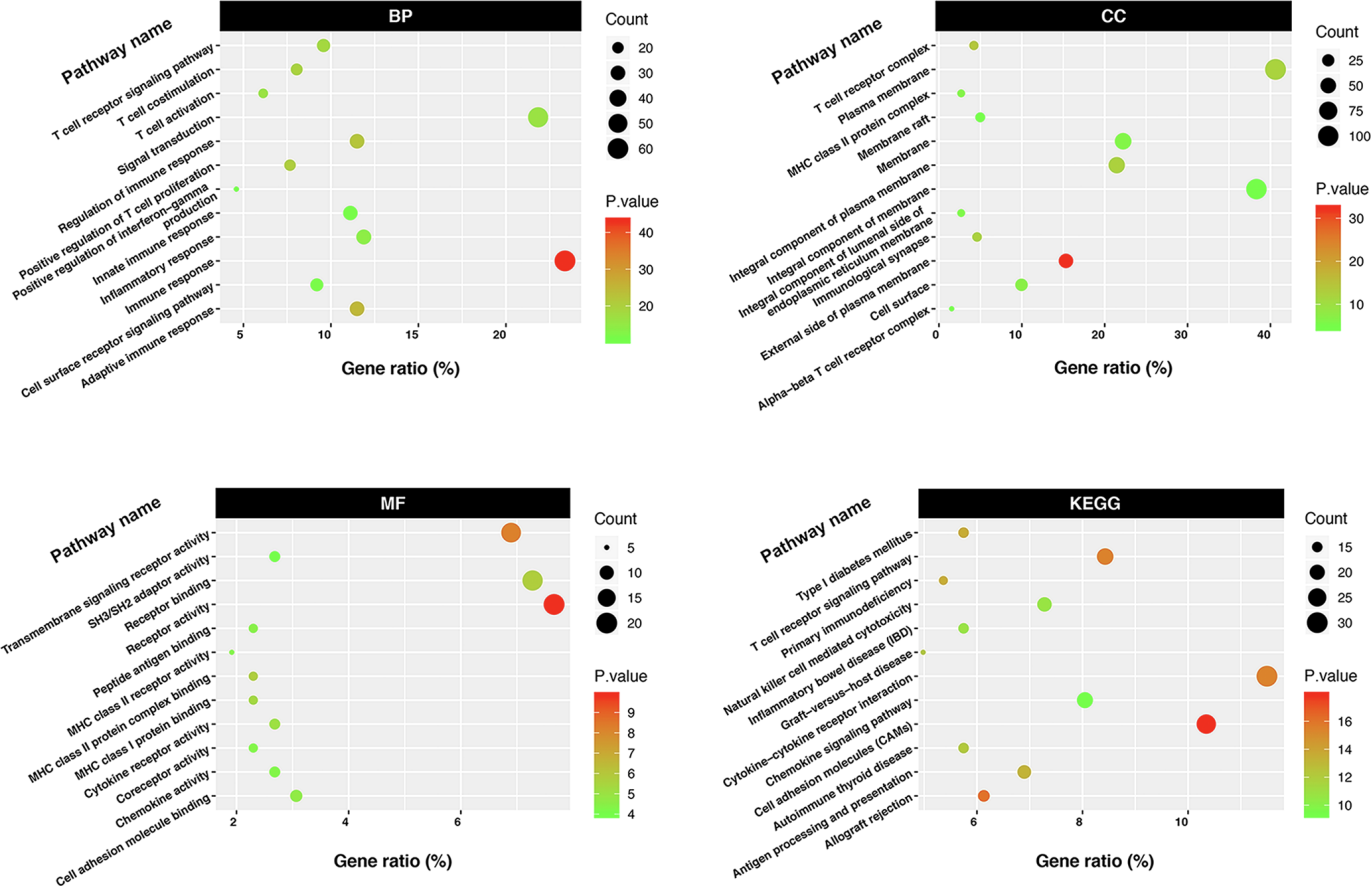
GO and KEGG enrichment analyses showed that CCL5/CCR5-related genes were mainly enriched in T-cell-related immune pathways.

**Figure 7:**
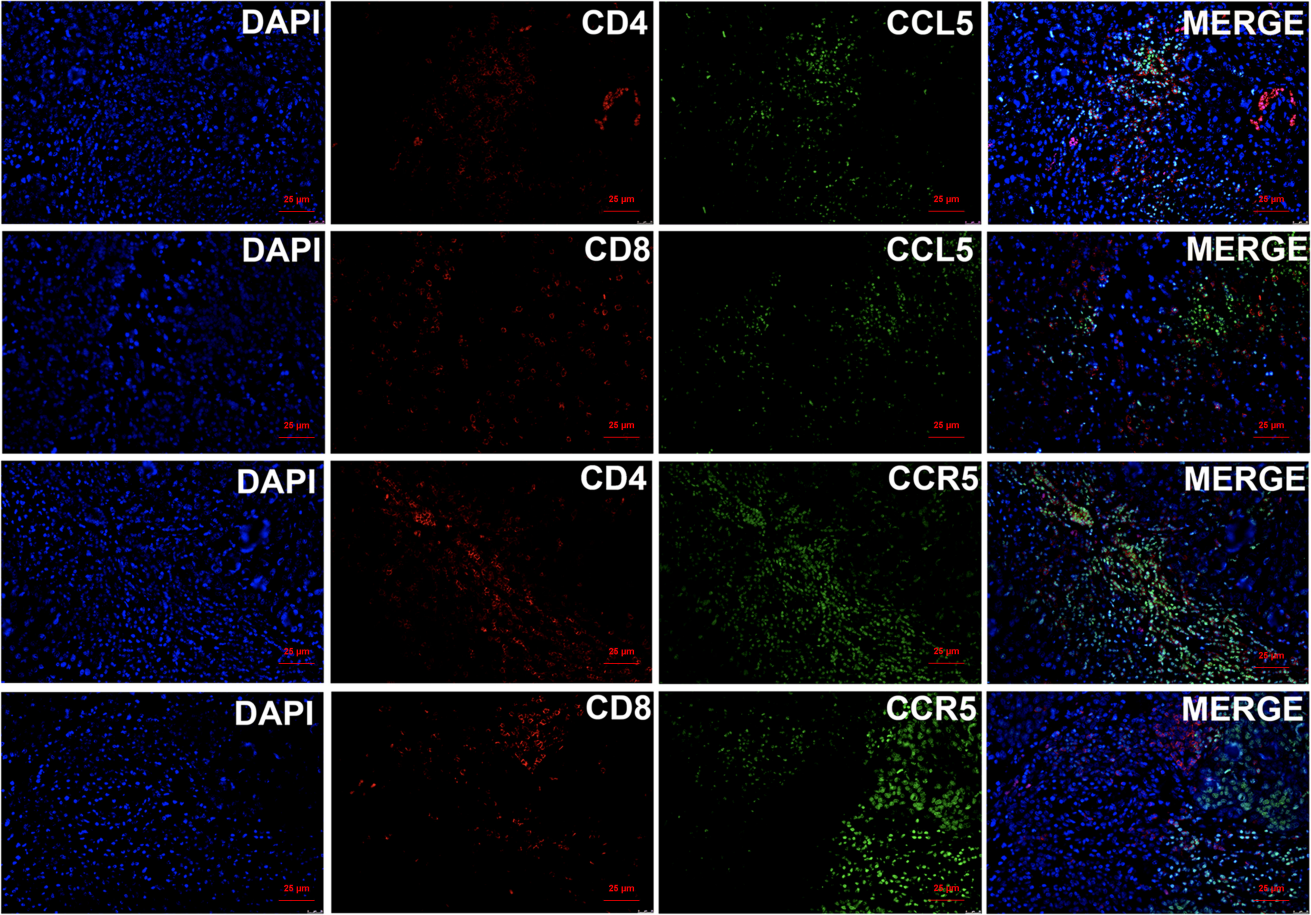
Immunofluorescence staining of CCL5/CCR5 and CD4/CD8 showed that CCL5/CCR5 is mainly expressed in CD4+ T cells but rarely in CD8+ T cells.

Based on the above results, we found that CCL5 and CCR5 jointly affect the biological behavior of breast cancer and the prognosis of breast cancer patients. To explore the mechanism by which CCL5/CCR5 promotes tumor metastasis, we conducted GO and KEGG enrichment analyses on CCL5- and CCR5-related genes, which showed that the genes enriched by CCL5/CCR5 were mainly enriched in immune-related signaling pathways, such as the T-cell receptor signaling pathway, immune response, T-cell costimulation, and T-cell activation. (**Figure 6**). We can conclude that T-cell-related immune pathways are significantly enriched in CCL5/CCR5-related genes. According to different surface markers, T cells can be divided into CD4+ and CD8+ subpopulations. We performed immunofluorescence staining of breast cancer tissues and found that CCL5 and CCR5 were mainly expressed in CD4+ T cells but rarely expressed in CD8+ T cells (**Figure 7**).

### CCL5/CCR5 promotes axillary lymph node metastasis of breast cancer *via* Treg cells

**Figure 8:**
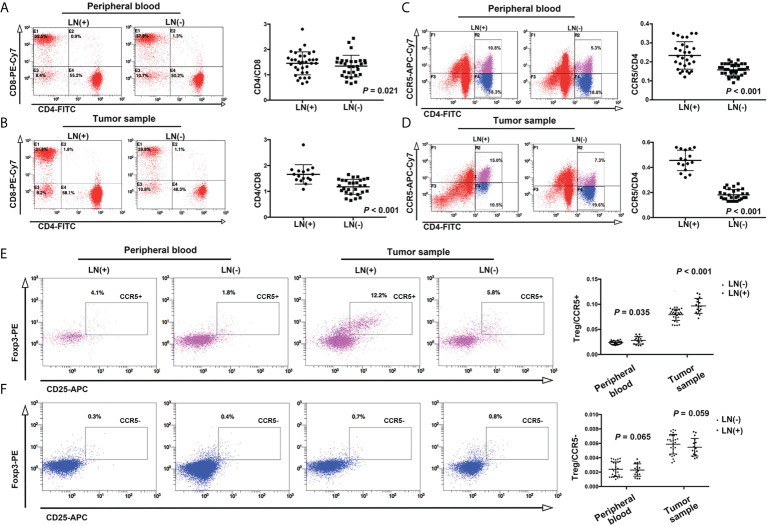
In the peripheral blood of patients with axillary lymph node metastasis of breast cancer, the ratios of CD4/CD8 and CCR5/CD4 were higher than in patients without lymph node metastasis **(A, C)**. This trend was more obvious in tumor tissues and cells **(B, D)**. **(E)** The proportion of CD4+CD25+Foxp3+ (Treg cells)/CCR5+ cells in peripheral blood and tumor tissue were higher in the lymph node metastasis group, while the proportion of Treg/CCR5- cells was not significantly different between the two groups **(F)**.

We analyzed peripheral blood and tumor tissue cells from breast cancer patients *via* flow cytometry to further explore the mechanism by which CCL5/CCR5 affects the progression of breast cancer. We found that the CD4+/CD8+ ratio was significantly higher in patients with axillary lymph node metastasis (1.54 ± 0.42) than in patients without axillary lymph node metastasis (1.29 ± 0.43) (**Figure 8A**, *P*=0.021). In addition, we found that the CCR5+/CD4+ ratio was higher in the axillary lymph node metastasis group (0.23 ± 0.07 vs. 0.16 ± 0.03, *P* < 0.001) (**Figure 8C**). In the analysis of tumor tissue cells, the tendencies were more obvious: the CD4+/CD8+ ratio in the metastasis group and nonmetastasis group was 1.66 ± 0.38 and 1.18 ± 0.29, respectively (**Figure 8B**, *P* < 0.001), and the CCR5+/CD4+ ratio was 0.46 ± 0.08 and 0.18 ± 0.04, respectively (*P* < 0.001) (**Figure 8D**).

Studies have reported that a decrease in the CD4+/CD8+ ratio is related to the occurrence of a variety of malignant tumors (23, 24), but in this study, we came to the opposite conclusion, which indicates that the role of CD4+CCR5+ T cells is completely opposite to that of tumor-infiltrating lymph node cells. Therefore, we speculate that the function of these T cells may be related to Treg cells.

Based on this assumption, we detected CD25 and Foxp3, which mark Treg cells. The results showed that an increase in the CD4+CD25+Foxp3+(Treg)/CCR5+ cell ratio was closely related to the invasiveness of tumors: the Treg/CCR5 ratio in the lymph node metastasis group and nonmetastasis group was 0.028 ± 0.007 and 0.024 ± 0.003, respectively, *P* = 0.035. In the tumor tissue lysate, this trend was more obvious: the Treg/CCR5 ratio in the lymph node metastasis group and nonmetastasis group was 0.097 ± 0.148 and 0.079 ± 0.112, respectively, *P* < 0.001(**Figure 8E**). However, there was no significant correlation between the Treg/CCR5- T-cell ratio and breast cancer invasiveness in either peripheral blood or tumor tissue cells (**Figure 8F**, *P* = 0.065 and 0.059, respectively).

## Discussion

This study analyzed the correlation between CCL5 and the invasion and metastasis of breast cancer. We found that CCL5 was positively correlated with axillary lymph node metastasis in breast cancer and that CCL5 was positively correlated with factors that predicted poor prognosis in breast cancer. Bioinformatics analysis revealed that the function of the CCL5/CCR5 signaling pathway was mainly concentrated in the T-cell-related immune pathway. Further flow cytometry analysis showed that CCL5/CCR5 may affect the prognosis of breast cancer through an increase in Treg/CCR5+ cells.

In this study, CCL5 in serum was related to the PR and clinical stage of breast cancer patients, and CCL5 in tumor tissue was related to ER, PR, nuclear grade, clinical stage and molecular subtyping. Furthermore, there was a linear positive correlation between CCL5 in serum and tumor tissue. It can be concluded that CCL5 in tumor tissue and serum is “released synchronously”. The CD4/CD8 of patients in the lymph node positive group increased significantly, and this result was more obvious in tumor tissue (14 fold) than that in peripheral blood, indicating that the main environment for CD4+ cells promoting cancer progression was in tumor tissue rather than peripheral blood, therefore, we speculate that tumor tissue may be the main source of CCL5; in other words, a considerable proportion of CCL5 in serum is produced by tumor tissue and released locally and throughout the whole body, which then affects the development of tumors in the whole body. The conclusion that CCL5 mainly comes from tumor cells has also been reported in previous studies (25, 26). A small amount of CCL5 produced by tumor cells is released outside the cell and binds to the corresponding receptor. On the one hand, it affects the biological function of tumor cells. On the other hand, CCL5 and its receptor complex may stimulate tumor cells to produce more CCL5, thus forming a positive feedback loop. We speculate that this is also the reason for the accumulation of a large amount of CCL5 in tumor tissues.

As a chemokine ligand, CCL5 needs to bind with the corresponding receptor protein to play a biological role. After analyzing the data of breast cancer patients in the GEO and TCGA databases, we found that CCL5 is likely to play a tumor-promoting role through CCR5. CCR5 is a cell membrane protein that is a member of the G protein-coupled receptor superfamily and is one of the main coreceptors for HIV to invade human cells. In recent years, with in-depth study of CCR5, its role in a variety of diseases, including HIV, influenza, insulin resistance, and tumors, has been confirmed (27–29). At present, some studies report that CCR5 and its ligand CCL5 act as a complex to link other molecules upstream or downstream to exert biological functions. For example, in colon cancer, tumor derived CCL5 can recruit fibroblasts through the CCR5/SLC25A24 signaling pathway to promote angiogenesis and collagen formation, thereby affecting the tumor microenvironment (25). In addition, studies have confirmed that CCL5 secreted by tumor cells recruits Treg cells through CCR5 to stimulate TGF-β to block the tumor killing function of CD8+ T cells, promoting tumor progression (26). In this study, CCL5 increased the apoptosis of CD8+ T cells, which was mediated by Tregs, consistent with the increase in CD4+/CD8+ T cells induced by CCL5 in our study. Recent studies have also confirmed that CCL5/CCR5 can affect the polarization of M2 macrophages through the MEK/STAT3 signaling pathway in luminal B breast cancer (30). In contrast to the above study, our study confirmed that CCL5/CCR5 may play a role in promoting metastasis in breast cancer, and the mechanism may be through an effect on Treg/CCR5+ cells.

In addition to tumor cells, CCL5 is also expressed on T cells (31). In the KEGG and GO enrichment analyses in this study, we found that CCL5 may affect the progression of breast cancer through T-cell-related immune pathways. Therefore, we speculate that in addition to the secretion of CCL5 by tumor cells, there is a mechanism similar to PD1/PDL1: CCL5 expressed by T cells combines with the CCR5 receptor on the surface of tumor cells to form the CCL5/CCR5 complex. The difference is that the targets of PD1/PDL1 are TILs, and tumor progression can be promoted by inactivation of TILs (32); however, our study confirmed that CCL5/CCR5 affects breast cancer progression and the survival of breast cancer patients by affecting the Treg/CCR5+ cell ratio. If this process is confirmed, it will hopefully become a potential molecular target for immunotherapy against breast cancer.

This study also has some limitations. Intervention in the activation of the CCL5/CCR5/Treg signaling pathway and the downstream effects need to be confirmed by further *in vivo* experiments.

## Conclusion

CCL5 affects the biological behavior of breast cancer and the prognosis of breast cancer patients through the CCR5/Treg signaling pathway, which is likely to be a potential therapeutic target for breast cancer.

## Data availability statement

The datasets presented in this study can be found in online repositories. The names of the repository/repositories and accession number(s) can be found in the article/supplementary material.

## Ethics statement

The studies involving human participants were reviewed and approved by medical ethics committee of Sichuan University. The patients/participants provided their written informed consent to participate in this study.

## Author contributions

(I) Conception and design: ZD and QL. (II) Administrative support: None. (III) Provision of study materials or patients: QL, XZ and LX. (IV) Collection and assembly of data: FL, HW and XZ. (V) Data analysis and interpretation: JQ, LX and FL. (VI) Manuscript writing: All authors. (VII) Final approval of manuscript: All authors.

## Funding

Key projects of Sichuan Provincial Health Commission (21PJ042); Incubation project of West China Hospital of Sichuan University (2022HXFH004); Natural Science Foundation of Sichuan Province (22NSFSC2361); Key research and development projects of Sichuan Provincial Department of science and technology (2021YFS0104); National Natural Science Foundation of China (No. 82100655) and Key research and development projects of Sichuan Science and Technology Department (22ZDYF1209).

## Conflict of interest

The authors declare that the research was conducted in the absence of any commercial or financial relationships that could be construed as a potential conflict of interest.

## Publisher’s note

All claims expressed in this article are solely those of the authors and do not necessarily represent those of their affiliated organizations, or those of the publisher, the editors and the reviewers. Any product that may be evaluated in this article, or claim that may be made by its manufacturer, is not guaranteed or endorsed by the publisher.
